# Nonlinear relationship between incidence of new-onset stroke and plasma atherosclerotic index in middle-aged and older adults

**DOI:** 10.3389/fneur.2025.1597616

**Published:** 2025-10-22

**Authors:** Qingqing Wang, Leiyong Zhao, Tianqi Zuo, Wei Peng

**Affiliations:** ^1^Medical Records Quality Control Office, Affiliated Hospital of Shandong University of Traditional Chinese Medicine, Jinan, China; ^2^Department of Acupuncture and Rehabilitation, Affiliated Hospital of Nanjing University of Chinese Medicine, Nanjing, China; ^3^The First Clinical College of Shandong University of Traditional Chinese Medicine, Jinan, China; ^4^Department of Neurology, Affiliated Hospital of Shandong University of Traditional Chinese Medicine, Jinan, China

**Keywords:** nonlinearity, AIP, stroke, CHARLS, cohort study

## Abstract

**Background:**

The arteriosclerosis index of plasma (AIP) is a sensitive biomarker that reflects characteristics of lipid metabolism and lipoprotein profiles, calculated as the logarithmic transformation of the ratio between fasting triglycerides (TG) and fasting high-density lipoprotein cholesterol (HDL-C). However, current evidence regarding the detailed relationship between AIP and the risk of stroke among middle-aged and elderly adults remains limited. Therefore, this study was conducted to comprehensively explore the link between AIP and the occurrence of new-onset stroke in middle-aged and elderly populations, aiming to provide an evidence-based foundation for stroke prevention and management.

**Methods:**

Data analyzed in this study were drawn from the China Health and Retirement Longitudinal Study (CHARLS), including 6,808 subjects aged ≥45 years without prior history of stroke. Logistic regression models and restricted cubic spline (RCS) analyses were employed to investigate the association between AIP values and stroke incidence. Subgroup analyses were conducted to examine potential sources of heterogeneity, and stratified analyses were performed to verify the robustness of the results.

**Results:**

Following extensive adjustment for potential confounding factors, logistic regression demonstrated that increased AIP was significantly associated with higher stroke incidence among middle-aged and elderly individuals (OR = 1.63, 95% CI: 1.09, 2.45, *p* = 0.02). RCS analysis further revealed a nonlinear dose–response relationship between AIP and stroke risk, identifying an inflection point at an AIP value of −0.02. Subgroup analyses revealed differences based on sex and age: a linear positive correlation was observed in males but not in females; similarly, no significant correlation appeared in individuals aged 45–59, while a positive correlation emerged in individuals aged 60 and above, with the association strengthening with age. Stratified analysis indicated no statistically significant interactions among strata.

**Conclusion:**

This study identifies a nonlinear, positive correlation between AIP and stroke incidence in middle-aged and elderly individuals, noting variations based on gender and age.

## Introduction

Stroke has become a substantial global health challenge due to its notably high incidence, ranking as the second most common cause of death and third leading cause of disability worldwide (1, 2). According to data from the Global Burden of Disease (GBD) study, between 1990 and 2019, global stroke prevalence rose by 85%, stroke-related mortality increased by 43%, and disability-adjusted life years (DALYs) attributable to stroke grew by 32% (3). Due to rapid population growth and aging, stroke incidence and related mortality have continuously risen, particularly among individuals aged 70 years and older (4). Moreover, the probability of stroke recurrence markedly increases with age (5). China, home to the world’s largest elderly population, accounts for approximately one-quarter of all new stroke cases annually and bears the highest stroke burden globally (6–8). This situation imposes substantial physical, psychological, and financial burdens on patients, while also placing considerable strain on healthcare and social services in China (9, 10).

Given the severe threat posed by stroke, early identification and management of risk factors are essential to mitigate stroke risk (11). Numerous studies have established a robust association between stroke and atherosclerosis (12, 13). Historically, evaluations of cardiovascular risk and atherosclerosis have predominantly focused on low-density lipoprotein cholesterol (LDL-C) and HDL-C measurements (14). Nonetheless, clinical observations reveal that achieving target LDL-C levels does not fully mitigate cardiovascular or cerebrovascular risks (15). Hypertriglyceridemia commonly coexists with decreased HDL-C concentrations, potentially exacerbating residual cardiovascular risks (16). The AIP, calculated as the logarithmic ratio of TG to HDL-C, precisely captures the lipid metabolism balance and presents considerable benefits compared to traditional lipid parameters (17). Research has consistently linked elevated AIP values to heightened risks of cardiovascular and cerebrovascular events, particularly major artery atherosclerotic ischemic stroke (18, 19). Nevertheless, prior studies have notable limitations: participants were often limited to specific populations such as obese individuals (20) and those with stage 0–3 cardiovascular-renal-metabolic (CKM) disease (21); data were collected from restricted communities (22) or individual medical institutions (23); and study designs were primarily cross-sectional (24), limiting their ability to establish a causal relationship between AIP and stroke risk. Currently, cohort studies exploring the association between AIP and stroke remain limited, and the possible nonlinear nature of this relationship and subgroup variations have not been thoroughly investigated. Therefore, investigating these relationships would address existing research gaps and provide empirical evidence for targeted intervention strategies and improved resource allocation in healthcare.

This study employs nationally representative data obtained from the CHARLS to examine the relationship between AIP and new-onset stroke risk. These results aim to provide novel insights into stroke etiology and assess the feasibility and effectiveness of using AIP as a predictive marker for stroke risk. Ultimately, the study aims to provide novel strategies for stroke detection and prevention.

## Methods

### Study population

CHARLS is a comprehensive longitudinal study designed to thoroughly examine the health and aging of Chinese individuals aged 45 and older (25). To achieve national representation, CHARLS employed a meticulous multistage stratified sampling design based on probability proportional-to-size, spanning 28 provinces, 150 counties/districts, and 450 villages/urban communities nationwide. Data collection encompassed 10,257 households, comprising 17,708 individual participants (26). The CHARLS database contains demographic, economic, pension, and health information with high reliability and validity, adequately representing the living and medical conditions of China’s middle-aged and elderly population (27). Recently, this database has become a critical resource for research on health-related topics.

**Figure 1:**
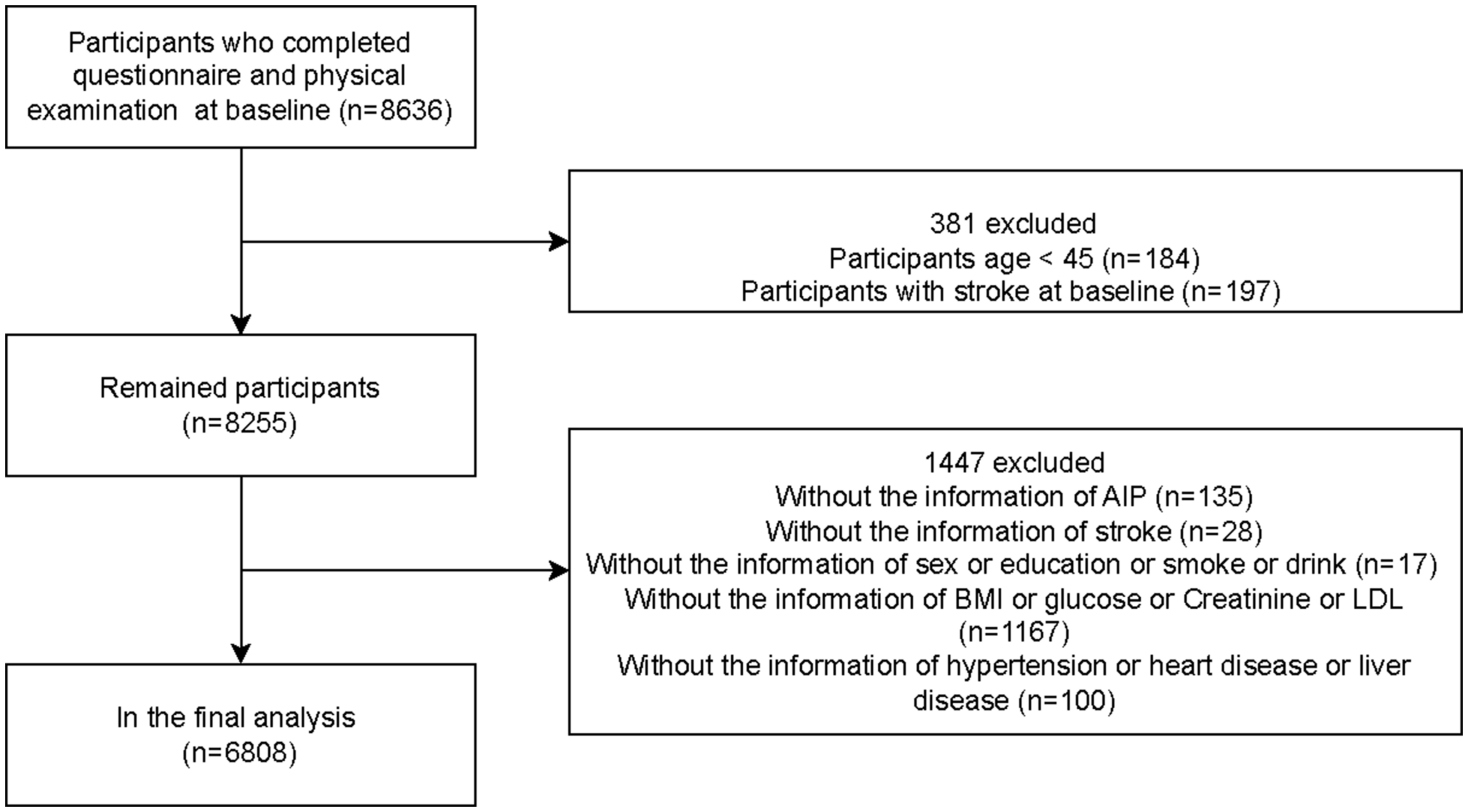
Flowchart for the selection of research subjects.

This study analyzed and compared baseline data from 2011 and follow-up data from 2018 in the CHARLS database, performing a seven-year longitudinal assessment. Participants were middle-aged and elderly individuals aged 45 or older in China. Ultimately, the analysis included 6,808 eligible subjects. The participant selection procedure is illustrated in Figure 1.

Ethical approval for this study was obtained from the Biomedical Ethics Committee of Peking University (approval number: RB000105211015).

### Data collection and analysis

#### Data collection

The study collected multidimensional data, including categorical variables such as sociodemographic characteristics, lifestyle factors, and chronic diseases, and continuous variables including laboratory test results and body mass index (BMI). Sociodemographic characteristics included age, sex (male or female), marital status (married or single), residence (urban or rural), and educational attainment (elementary school or below, high school, vocational school or above). Lifestyle factors consisted of smoking status (never, former, current) and drinking habits (non-drinker, drinking less than once a month, drinking more than once a month). Data on chronic diseases were also collected, including hypertension, diabetes, heart disease, and liver disease. Laboratory measurements included glucose, creatinine, urea, low-density lipoprotein (LDL), and high-density lipoprotein (HDL). BMI was calculated as weight (kg) divided by height squared (m^2^). The CHARLS database implemented strict quality control measures during data collection, ensuring accuracy and reliability.

#### Assessment and validation of new-onset stroke

Identification of new-onset stroke relied on participants’ responses to the questionnaire item: “Have you been diagnosed with stroke by a doctor (including cerebral infarction and cerebral hemorrhage)?” (28–30). Participants answering “yes” were classified as stroke patients. Individuals diagnosed with stroke in 2011 were excluded. Patients diagnosed after 2011 and fulfilling the criteria for new-onset stroke during the 2018 follow-up period were included. Although stroke diagnoses depended primarily on self-reported physician diagnoses, previous validation studies showed a sensitivity of 81.3% for initial stroke events, with high consistency with medical records, confirming its accuracy in similar large-scale surveys (31–33).

#### Definitions and calculations

Hypertension was assessed based on participants’ self-reported physician-confirmed history, current antihypertensive medication use, or blood pressure measurements. Blood pressure measurements were performed three consecutive times under standardized protocols by trained examiners, with the mean of these readings utilized for analysis. Information about liver disease and cardiovascular conditions was acquired from participant-reported histories of physician diagnoses or medication usage related to these illnesses. Diabetes was determined by participants’ self-reported medical diagnoses confirmed by physicians, use of insulin or oral antidiabetic medications, glycated hemoglobin (HbA1c) values of at least 6.5%, or fasting blood glucose measurements of at least 126 mg/dL. The logarithmic transformation of the triglyceride-to-high-density lipoprotein cholesterol ratio [log(TG/HDL-C)] was employed to compute the Arteriosclerosis Index of Plasma (AIP) (34).

#### Data analysis

All statistical analyses were conducted using R statistical software (version 4.4.2). Categorical variables were presented as percentages, whereas continuous variables were expressed as mean ± standard deviation (mean ± SD). A multivariate-adjusted binary logistic regression model was established (35): The crude model was unadjusted. Model 1 adjusted for demographic characteristics (age, sex, residence, education, marital status) and lifestyle factors (smoking and drinking habits) to evaluate the strength of association independent of clinical factors. Model 2 further adjusted for chronic diseases (hypertension, heart disease, diabetes, liver disease) based on Model 1, aiming to control confounding from pre-existing conditions. Model 3 included laboratory indicators (glucose, creatinine, urea, LDL, HDL) and BMI in addition to variables from Model 2, to assess the robustness of the exposure association after controlling for physiological status. This incremental approach sequentially isolated potential confounding across multiple levels (36, 37). Furthermore, a restricted cubic spline (RCS) model was employed to analyze the dose–response relationship between AIP and stroke incidence. Considering clinically significant variables such as age, sex, and chronic diseases, relevant exploratory subgroup analyses were subsequently conducted (38–40). Findings were presented as odds ratios (OR) accompanied by 95% confidence intervals (CIs), and statistical significance was defined as a *p*-value below 0.05.

## Results

### Basic information on participants

Table 1 presents baseline characteristics of the 6,808 middle-aged and older participants. Of these, 3,042 (44.68%) were male, and 3,766 (55.32%) were female. During follow-up, 543 cases (7.98%) of new-onset stroke were identified, with 46.78% occurring among males and 53.22% among females. Further analysis of participants by age group indicated that the largest proportion was aged 45–59 (57.36%), followed by those aged 60–69 (30.10%) and ≥70 years (12.54%). Most participants were married (89.25%), resided in rural areas (65.98%), and had primary education (69.79%). Stroke incidence was associated with age, marital status, smoking, BMI, blood glucose, creatinine, HDL levels, hypertension, diabetes, heart disease, and liver disease (*p* < 0.05). Clinical characteristics stratified by AIP values (see Supplementary Table S1) demonstrated statistically significant correlations with age, sex, marital status, education, residence, smoking status, drinking habits, BMI, glucose, creatinine, HDL, heart disease, hypertension, and diabetes (*p* < 0.05).

**Table 1:** The baseline characteristics of participants.

Variable	Total (*n* = 6,808)	No (*n* = 6,265)	Yes (*n* = 543)	Statistic	*P*-value
Age**_**group				36.05	<0.0001
45 ~ 59	3,905 (57.36)	3,656 (58.36)	249 (45.86)		
60 ~ 69	2,049 (30.10)	1,855 (29.61)	194 (35.73)		
70+	854 (12.54)	754 (12.04)	100 (18.42)		
Marital**_**status				4.77	0.03
Married	6,076 (89.25)	5,607 (89.50)	469 (86.37)		
Single	732 (10.75)	658 (10.50)	74 (13.63)		
Smoke				6.57	0.04
Current	2,005 (29.45)	1,847 (29.48)	158 (29.10)		
Former	546 (8.02)	487 (7.77)	59 (10.87)		
Never	4,257 (62.53)	3,931 (62.75)	326 (60.04)		
Hypertension				164.75	<0.0001
No	5,168 (75.91)	4,879 (77.88)	289 (53.22)		
Yes	1,640 (24.09)	1,386 (22.12)	254 (46.78)		
DM				41.92	<0.0001
No	6,402 (94.63)	5,924 (95.16)	478 (88.52)		
Yes	363 (5.37)	301 (4.84)	62 (11.48)		
Heart.disease				45.82	<0.0001
No	6,083 (89.35)	5,645 (90.10)	438 (80.66)		
Yes	725 (10.65)	620 (9.90)	105 (19.34)		
Liver.disease				11.67	<0.001
No	6,593 (96.84)	6,081 (97.06)	512 (94.29)		
Yes	215 (3.16)	184 (2.94)	31 (5.71)		
AIP	−0.02 ± 0.33	−0.02 ± 0.33	0.06 ± 0.32	−5.72	<0.0001
BMI	23.63 ± 3.90	23.55 ± 3.88	24.46 ± 4.05	−5.04	<0.0001
Glucose	108.64 ± 31.77	108.09 ± 31.07	114.94 ± 38.47	−4.03	<0.0001
Creatinine	0.77 ± 0.18	0.77 ± 0.18	0.79 ± 0.18	−2.60	<0.01
HDL	51.54 ± 15.18	51.76 ± 15.20	48.93 ± 14.71	4.28	<0.0001

### Association between AIP and stroke

This study evaluated the relationship between AIP (independent variable) and stroke incidence (dependent variable) using multivariate logistic regression models. Compared with participants in the lowest AIP quartile, those in the second, third, and fourth quartiles showed an increased stroke risk of 55% (OR = 1.55, 95% CI [1.17, 2.05], *p* = 0.002), 86% (OR = 1.86, 95% CI [1.42, 2.44], *p* < 0.0001), and 106% (OR = 2.06, 95% CI [1.58, 2.69], p < 0.0001), respectively.

To control for potential confounding factors, a stepwise adjustment approach was employed. After adjusting for age, sex, education, residence, marital status, smoking status, drinking habits, hypertension, diabetes, heart disease, liver disease, BMI, glucose, creatinine, urea, HDL, and LDL in the final model, stroke incidence remained significantly elevated by 45% (OR = 1.45, 95% CI [1.06, 1.98], *p* = 0.02), 57% (OR = 1.57, 95% CI [1.11, 2.22], *p* = 0.01), and 63% (OR = 1.63, 95% CI [1.09, 2.45], *p* = 0.02) across the second, third, and fourth quartiles of AIP, respectively. Additionally, when analyzed as a continuous variable, each interquartile range (IQR) increase in AIP corresponded to a 27% greater stroke risk (OR = 1.27, 95% CI [1.04, 1.54]). Similarly, each standard deviation (SD) increase in AIP was associated with a 20% higher stroke risk (OR = 1.20, 95% CI [1.03, 1.39]) (Table 2).

**Table 2:** The association between AIP and incidence of stroke across various models.

Character	Crude model		Model 1		Model 2		Model 3	
	95%CI	*P*	95%CI	*P*	95%CI	*P*	95%CI	*P*
Stroke~AIP	2.07 (1.61, 2.68)	<0.0001	2.25 (1.73, 2.92)	<0.0001	1.71 (1.30, 2.25)	<0.001	1.74 (1.10, 2.74)	0.02
Stroke~AIPQ								
Q1	ref		ref		ref		ref	
Q2	1.55 (1.17, 2.05)	0.002	1.58 (1.19, 2.10)	0.001	1.49 (1.12, 1.98)	0.01	1.45 (1.06, 1.98)	0.02
Q3	1.86 (1.42, 2.44)	<0.0001	1.93 (1.47, 2.54)	<0.0001	1.64 (1.24, 2.17)	<0.001	1.57 (1.11, 2.22)	0.01
Q4	2.06 (1.58, 2.69)	<0.0001	2.2 (1.67, 2.89)	<0.0001	1.73 (1.31, 2.29)	<0.001	1.63 (1.09, 2.45)	0.02
*p* for trend		<0.0001		<0.0001		<0.001		0.04
Stroke~AIP_IQR	1.37 (1.22, 1.52)	<0.0001	1.41 (1.26, 1.58)	<0.0001	1.26 (1.12, 1.41)	<0.001	1.27 (1.04, 1.54)	0.02
Stroke~AIP_SD	1.27 (1.17, 1.38)	<0.0001	1.3 (1.20, 1.42)	<0.0001	1.19 (1.09, 1.30)	<0.001	1.2 (1.03, 1.39)	0.02

Crude model: Not adjusted.

Model 1: AIP, Age, Sex, Education, Marital_status, Residence, Drink, Smoke.

Model 2: AIP, Age, Sex, Education, Marital_status, Residence, Drink, Smoke, Heart.disease, Hypertension, DM, Liver.disease.

Model 3: AIP, Age, Sex, Education, Marital_status, Residence, Drink, Smoke, Heart.disease, Hypertension, DM, Liver.disease, BMI, Glucose, Creatinine, Urea, HDL, LDL.

**Figure 2:**
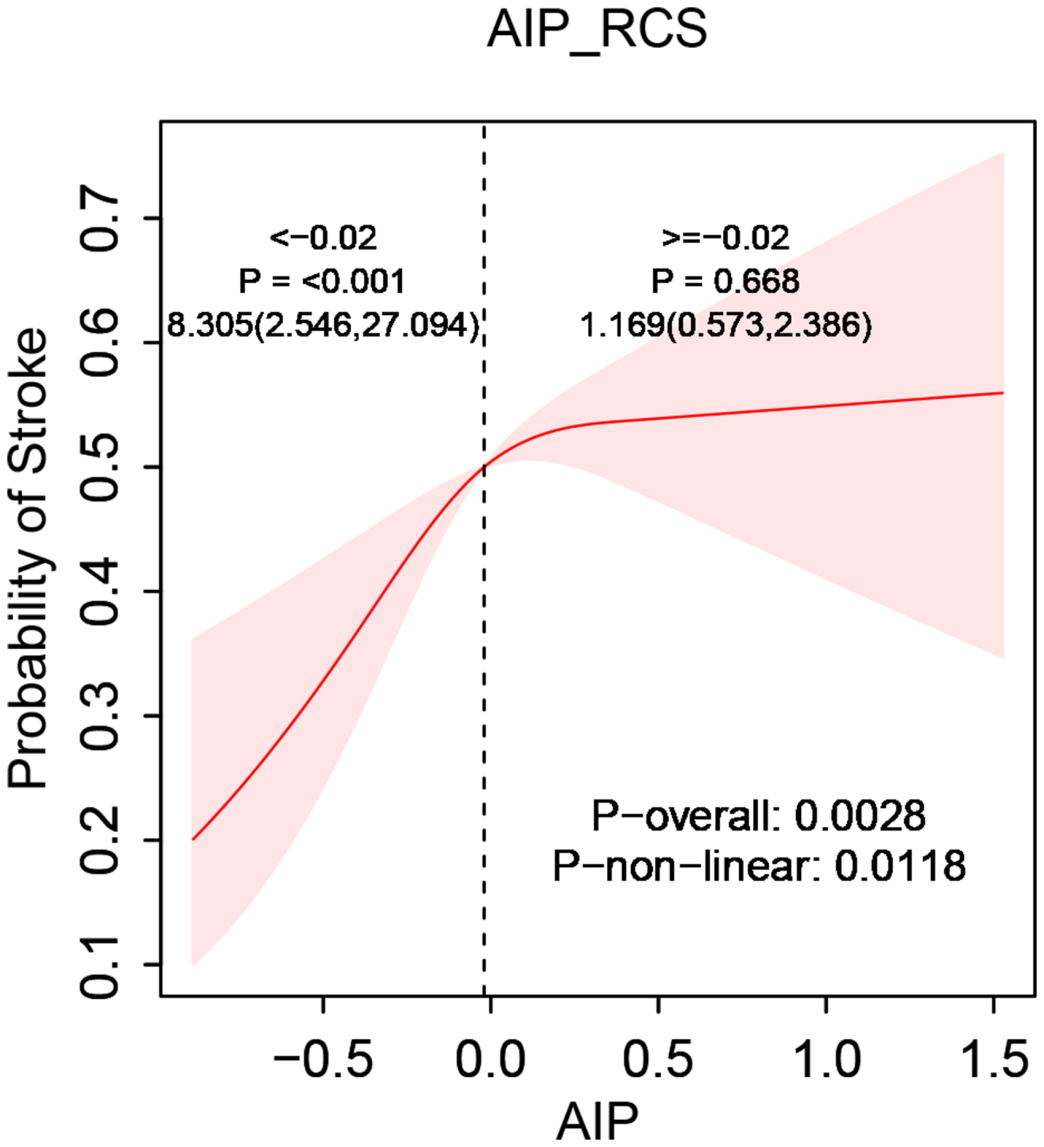
The relationship between AIP and stroke risk. Models were tuned for age, sex, residence, marital status, smoking, drink consumption, hypertension, diabetes, heart disease, liver disease, body mass index, glucose levels, creatinine, urea, HDL, and LDL.

After comprehensive adjustment for confounders, RCS regression further clarified the relationship between AIP and stroke incidence, highlighting a nonlinear positive association (p-nonlinear < 0.05). Threshold analysis pinpointed an inflection at an AIP level of −0.02, notably lower than the traditional “low-risk” criterion (AIP < 0.11) previously defined in literature (41). This finding suggests that even within the conventional “normal” AIP range, stroke risk sharply increased as AIP rose from its lowest value up to −0.02 (OR = 8.305, 95% CI [2.546, 27.094]). After surpassing the inflection point, the risk increase rate slowed significantly (OR = 1.169, 95% CI [0.573, 2.386]). Consequently, an AIP of −0.02 represented not merely a statistical inflection point but potentially a “high-risk-normal” state within the normal reference range. Clinically, this threshold might indicate the need for intensified monitoring or early intervention even when AIP remains conventionally normal (Figure 2).

**Figure 3:**
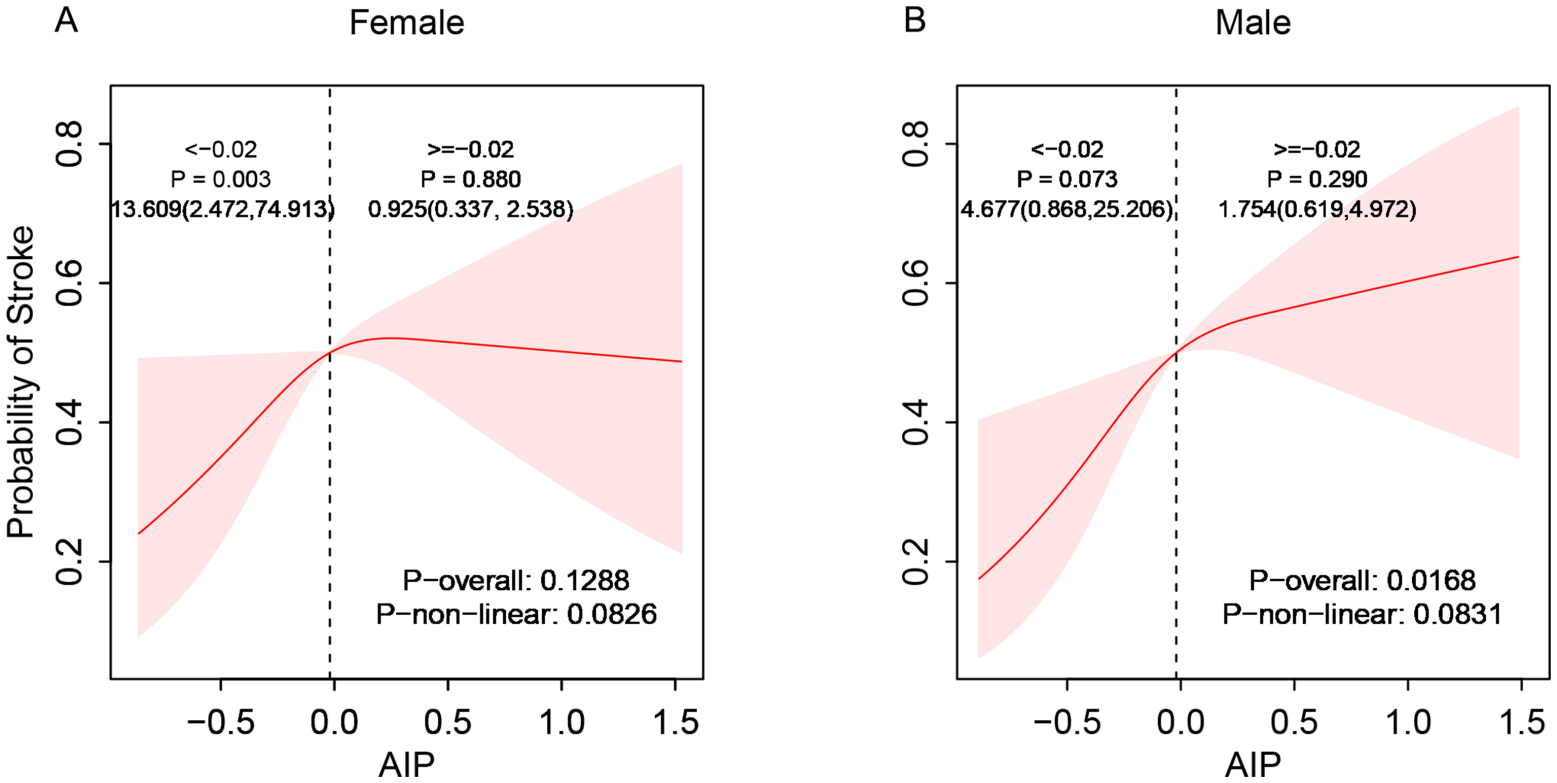
Sex-stratified association between AIP and stroke incidence rates (**A**: female; **B**: male).

**Figure 4:**
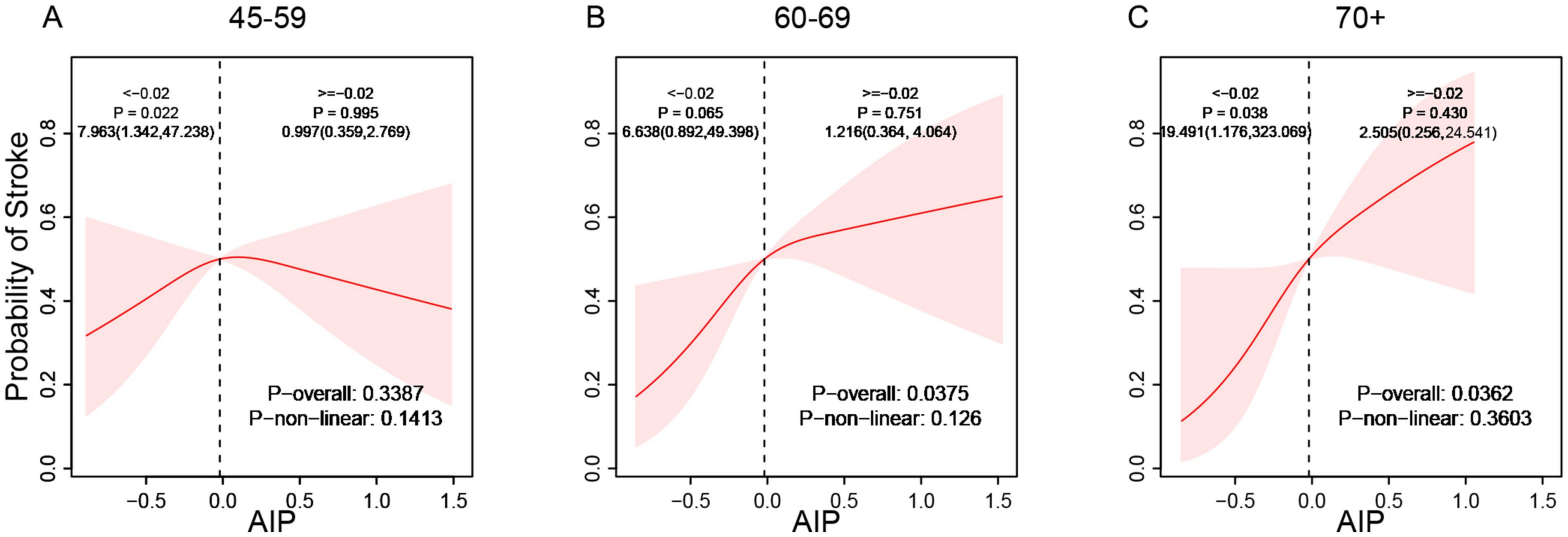
Age-stratified association between AIP and the incidence of stroke (**A**: 45–59, **B**: 60–69, **C**: 70+).

Subgroup analyses demonstrated considerable effect modification regarding the AIP-stroke relationship. Stratified by gender, the correlation between AIP and stroke exhibited a significant linear positive trend in males (p-overall < 0.05) but no statistical significance in females (p-overall > 0.05, Figure 3). Age-stratified analysis showed no significant relationship between AIP and stroke risk among individuals aged 45–59 (p-overall > 0.05). However, a linear positive correlation appeared in participants aged 60–69 and those aged ≥70 years, with correlation strength increasing with age (p-overall < 0.05, Figure 4). These findings indicate enhanced predictive capacity of AIP for stroke risk among males and individuals aged ≥60, supporting its potential as a biomarker for targeted stroke risk stratification.

### Stratified analysis

**Figure 5:**
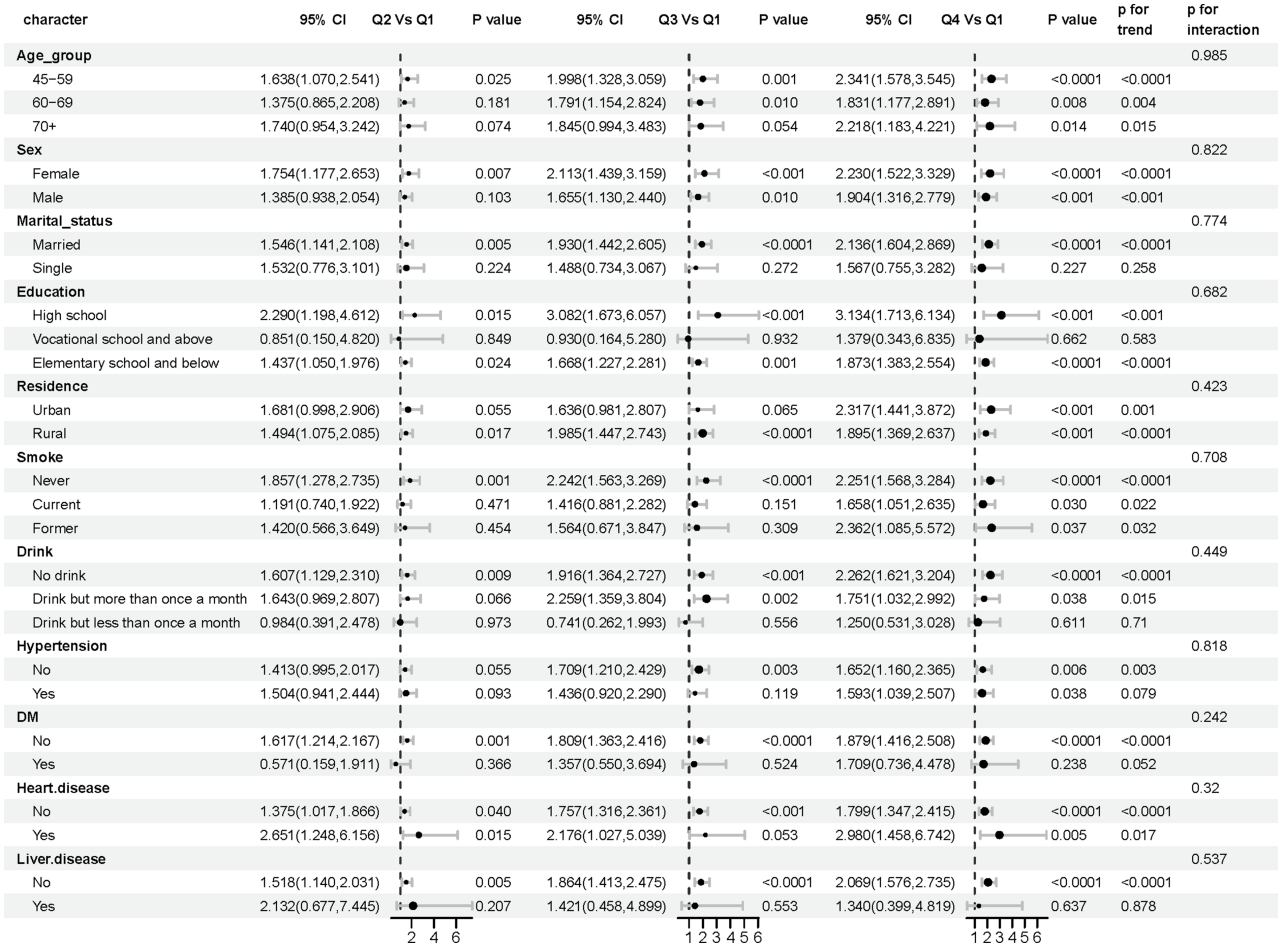
Forest plot: subgroup analysis of AIP and stroke risk elements. In subgroup analyses differentiated by age, sex, marital status, education, residence, smoking, drinks, hypertension, diabetes, heart disease, and liver disease, the models had no adjustment for these variables.

To evaluate the reliability and consistency of the identified relationship between stroke and AIP, stratified subgroup analyses were conducted and summarized in a forest plot (Figure 5). The results demonstrated consistent associations across all subgroups stratified by age, sex, marital status, education, residence, smoking status, drinking habits, hypertension, diabetes, heart disease, and liver disease, and no significant interaction terms were detected (interaction *p*-values > 0.05), further confirming the robustness of the relationship between AIP and stroke occurrence.

## Discussion

This large-scale investigation employed a seven-year longitudinal cohort design to explore the association between AIP and the risk of incident stroke. A total of 6,808 individuals aged 45 years or older from the CHARLS database, all free of stroke at baseline, were included. After comprehensive adjustment for potential confounders, the analysis demonstrated a significant positive association between elevated AIP levels and increased stroke incidence. Notably, using RCS analysis, this study first demonstrated that the association was a nonlinear dose–response relationship. Additionally, AIP exhibited stronger predictive performance among males and older populations.

The study identified a clear inflection point in the relationship between AIP and stroke risk. This inflection point (−0.02) was significantly below the conventional “low-risk” threshold (AIP < 0.11) (41), indicating that cerebrovascular harm from dyslipidemia might begin even within the “normal range.” As AIP approached −0.02, stroke risk increased markedly, suggesting that dysregulation of lipid metabolism became the predominant risk factor during this period (42, 43). The slowing of stroke risk beyond this inflection point implied the emergence of other pathogenic mechanisms, such as inflammatory responses and oxidative stress, which reduced the relative impact of lipid abnormalities (44). This inflection point might represent a critical pathophysiological transition, challenging conventional definitions of the “normal range” for AIP and requiring a reevaluation of its safety thresholds.

Notably, the predictive capacity of AIP demonstrated significant subgroup heterogeneity. This correlation was considerably stronger among males than females, possibly due to the regulatory role of sex hormones (45). After age 40, men experience a gradual decline in testosterone (46), potentially exacerbating AIP-related pathogenic effects by influencing lipid metabolism and inflammation. On one hand, testosterone played a crucial regulatory role in lipid metabolism, with lower testosterone resulting in elevated TG and LDL-C levels, decreased HDL-C, and subsequently increased AIP (47). On the other hand, testosterone exhibited anti-inflammatory properties, and its deficiency could lead to increased inflammation, disrupted lipid metabolism, elevated oxidized LDL (oxLDL), and thus higher AIP levels (48). Furthermore, smoking and alcohol consumption could modify the relationship between testosterone and AIP by disrupting endocrine balance and increasing oxidative stress (47). These factors collectively made men more susceptible to stroke risk related to increased AIP levels, suggesting that elevated AIP among middle-aged and elderly men might serve as an early indicator of metabolic disturbances, warranting targeted clinical interventions.

Furthermore, the predictive value of AIP for stroke increased notably with age. This finding illustrated the complex interplay between aging and lipid metabolism in stroke pathogenesis. From a pathophysiological viewpoint, increasing vascular dysfunction in older adults significantly exacerbated AIP-related risk. With aging, cerebral vessels experienced changes, including reduced arterial elasticity, vascular wall thickening, endothelial dysfunction, and diminished cerebral blood flow autoregulation (49, 50). These factors collectively contributed to vascular aging. In this context, the atherogenic lipid profile indicated by elevated AIP interacted with age-associated chronic inflammation, accelerating atherosclerosis and plaque instability and reducing compensatory mechanisms against ischemic injury (51, 52). Furthermore, age-related worsening of insulin resistance and hepatic lipid metabolism impairment enhanced the sensitivity of AIP as a marker of metabolic imbalance (53). Thus, even slight AIP elevations in individuals aged ≥60 years should be considered high-risk conditions.

These findings have substantial clinical and public health implications. First, the identified inflection point (AIP = −0.02) redefines baseline stroke risk assessment. Establishing this point as a preliminary warning threshold for stroke risk evaluation is recommended, especially for high-risk groups, such as those with cerebral small-vessel disease, family stroke history, or other vascular risk factors. Clinically, patients whose AIP approaches or exceeds this threshold, even within conventional low-risk ranges, should receive intensified monitoring and targeted lifestyle modifications. Second, identified subgroup heterogeneity supports tailored preventive strategies. Clinical practice should particularly emphasize AIP assessment in males over 40, considering elevated AIP (> − 0.02) as a crucial indicator for stroke risk and possibly including testosterone evaluations. Additionally, integrating AIP into standard screening protocols for individuals aged ≥60 years is advised. AIP acts as a sensitive risk amplifier; even modest increases necessitate rigorous monitoring and proactive cardiovascular prevention. In summary, this study proposes a precise biological threshold (AIP = −0.02) suitable for clinical risk assessment, advocating dynamic, stratified, and proactive risk management. This strategy could optimize resource allocation, facilitate early identification, and enable effective intervention in high-risk groups, thereby enhancing primary stroke prevention efficacy.

Despite these significant findings, several limitations warrant consideration. First, stroke outcomes partly depended on self-reported data. Post-event verification could result in misclassification or underreporting. Second, despite adjustments for numerous established risk factors, residual confounding, especially from unmeasured or unknown variables, could not be completely ruled out due to observational study limitations. Third, the study exclusively included a Chinese population, limiting the generalizability of findings to other ethnic groups. Fourth, participants were exclusively middle-aged or elderly (≥45 years), excluding younger age groups. Thus, generalization to broader populations requires caution. Future research should employ more precise outcome measures, comprehensive strategies to manage confounding variables, and validate results across diverse cohorts. Additionally, investigating the biological mechanisms linking AIP to stroke risk is needed. Nonetheless, this study holds substantial clinical value as the first investigation identifying a nonlinear association between AIP and stroke in middle-aged and elderly Chinese populations. Its findings provide new insights into using AIP for primary stroke prevention and risk management.

## Conclusion

Drawing on CHARLS data, this cohort study is the first to identify a nonlinear association between AIP and stroke risk among middle-aged and older Chinese adults, revealing a distinct inflection point at −0.02. This association showed stronger predictive value in males and older individuals. These results support integrating AIP into stroke risk models for high-risk groups, enabling early recognition of at-risk individuals and timely implementation of targeted interventions. Future studies should further validate the clinical utility of this threshold in diverse and multi-center populations and explore underlying molecular mechanisms. These findings offer a foundation for enhancing clinical stroke-risk management and revising consensus guidelines.

## Data Availability

Publicly available datasets were analyzed in this study. This data can be found here: https://charls.pku.edu.cn.
